# Two New Triterpenoid Saponins with Antifungal Activity from *Camellia sinensis* Flowers

**DOI:** 10.3390/ijms26031147

**Published:** 2025-01-28

**Authors:** Jian-Fa Zong, Zhi-Bo Hong, Zi-Hui Hu, Ru-Yan Hou

**Affiliations:** National Key Laboratory for Tea Plant Germplasm Innovation and Resource Utilization, Anhui Provincial Key Laboratory of Food Safety Monitoring and Quality Control, Joint Research Center for Food Nutrition and Health of IHM, Anhui Agricultural University, Hefei 230036, China; zonjfa@ahau.edu.cn (J.-F.Z.); hzb1285688@163.com (Z.-B.H.); 18779650950@163.com (Z.-H.H.)

**Keywords:** triterpenoid saponins, camsinsaponins, *Camellia sinensis*, antifungal, *Candida*

## Abstract

Two new triterpenoid saponins, namely camsinsaponins A and B (**1**, **2**), along with two known congeners (**3**, **4**) were isolated from *Camellia sinensis* flowers. Their structures were determined by extensive spectroscopic data. All compounds were assessed for antifungal bioactivity against *Candida albicans*, *Candida glabrata*, and *Candida tropicalis*. Compounds **1**–**4** showed excellent inhibitory effects. Notably, in regard to compounds **1** and **2**, their MIC values against *C. albicans* were close to those of the positive control, fluconazole. Furthermore, the inhibitory elements of compounds **1**–**4** on *C. glabrata* were better than those of fluconazole. The aforementioned findings offer valuable insights for future development of novel therapeutic strategies against drug-resistant infections.

## 1. Introduction

*Candida* species are common pathogenic fungi that have caused a large number of human fungal infections worldwide. It has been reported that Candida is the fourth leading cause of hospital-acquired bloodstream infections [1,2]. *Candida albicans*, as one of the most common pathogenic fungi, can cause various diseases that range from superficial to life-threatening, especially in immunocompromised patients [3,4,5]. The occurrence of invasive *Candida* infections is often associated with the extensive use of corticosteroids, prolonged use of broad-spectrum antibiotics and impaired immune function. Currently, the types of antifungal drugs available are limited, primarily including azoles, polyenes, and echinocandins [6]. However, studies have shown that the widespread and extensive use of these drugs may lead to severe fungal resistance [2]. Additionally, the majority of antifungal agents exhibit limited targeting specificity and are often associated with significant adverse effects, such as nephrotoxicity and hepatotoxicity [7,8]. Therefore, the development of novel antifungal agents is urgently needed.

**Figure 1:**
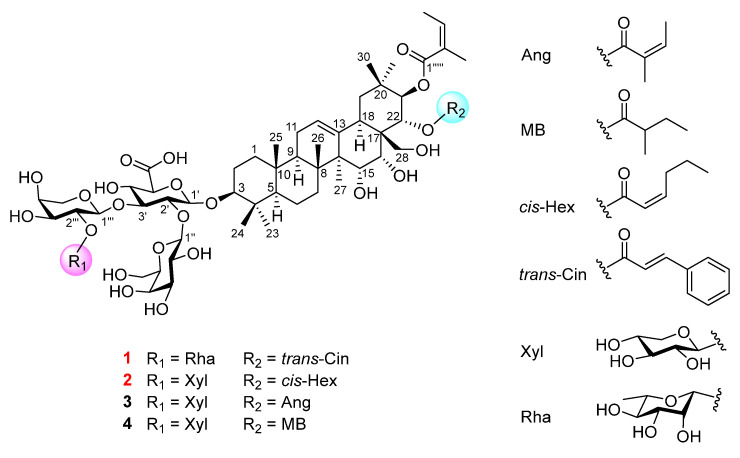
Structures of compounds **1** and **2**.

Natural products are renowned for their unique chemical diversity and biological activity, and they have been widely utilized in the treatment of various diseases for centuries. According to statistics, over half of the new drugs approved by the FDA between 1981 and 2019 were derived from natural products [9]. Triterpenoid saponins derived from *Camellia* plants exhibit highly diverse and complex structures, along with a wide range of beneficial biological activities [10]. These compounds have been widely utilized in the agricultural, pharmaceutical and cosmetic industries [11] due to their antimicrobial [12], antioxidant [13], antifungal [14], antitumor [15,16,17,18,19], biopesticide and insecticidal activity [20]. Previous studies have shown that triterpenoid saponins exhibited good inhibitory activity against *Candida albicans* [14]. Two saponins were evaluated against *C. albicans* ATCC10231 through physiological and biochemical examinations, morphological characteristics and transcriptome analyses. In our previous research, we have developed a new method for quantifying saponins in *Camellia* plants based on their hemolytic activity [21]. Then, we found that there are abundant saponins in *C. sinensis* flowers. As a new resource food in China, the flowers of *C. sinensis* exhibit substantial potential for utilization in the food and pharmaceutical sectors. Therefore, to better utilize the resources of *C. sinensis* flowers and investigate their potential as a source of natural antifungal agents, we performed isolation, identification and antifungal activity assessments for saponins extracted from *C. sinensis* flowers. Herein, four triterpenoid saponins were isolated and characterized (Figure 1), and we evaluated their inhibitory effects against *Candida albicans*, *Candida glabrata* and *Candida tropicalis*. More significantly, compounds **1**–**4** showed excellent inhibitory effects, especially in regard to *Candida albicans*.

## 2. Results and Discussion

### 2.1. Structural Identification

**Figure 2:**
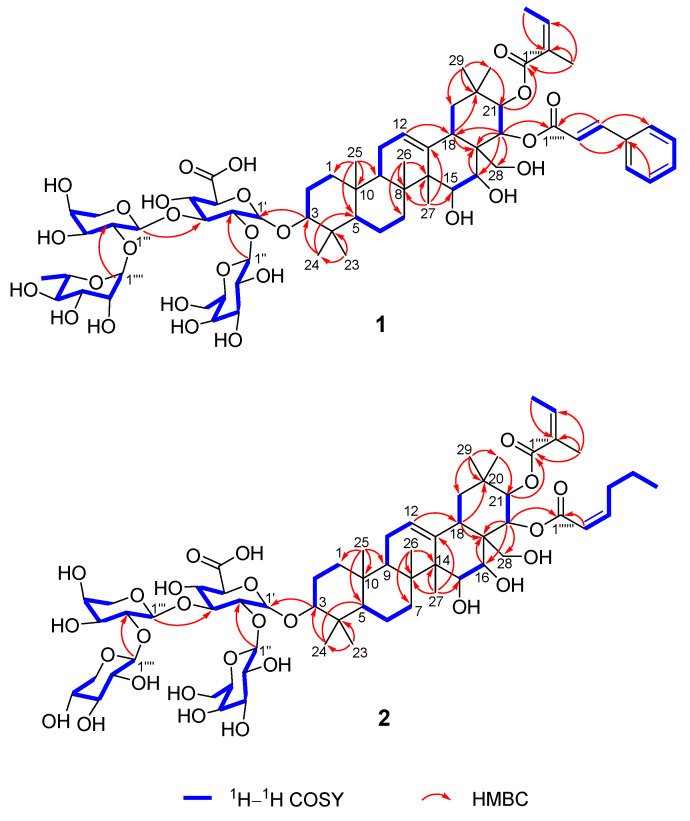
^1^H−^1^H COSY and key HMBC correlations of compounds **1** and **2**.

Compound **1** was isolated as a white amorphous powder. The HR-ESIMS negative ion at *m*/*z* 1333.6220 [M − H]^−^ (calcd. for C_67_H_97_O_27_, 1233.6217) revealed the presence of nineteen degrees of unsaturation. Its IR spectrum absorption peaks were consistent with the existence of hydroxy (3431 cm^−1^), carbonyl (1708 cm^−1^) and olefinic (1633 cm^−1^) functional groups. The ^1^H NMR data (Table 1) of **1** displayed ten methyl groups (*δ*_H_ 0.84, s, H_3_-25; 1.02, s, H_3_-26: 1.15, s, H_3_-29; 1.18, s, H_3_-24; 1.26, s, H_3_-23; 1.37, s, H_3_-30; 1.52, d, *J* = 4.2 Hz, H-6′′′′; 1.87, s, H_3_-27; 2.03, overlapped, H_3_-4′′′′′/H_3_-5′′′′′), four olefinic protons (*δ*_H_ 5.54, br s, H-12; 5.88, dq, *J* = 7.2 Hz, H-3′′′′′; 6.31, d, *J* = 16.2 Hz, H-2′′′′′′; 7.83, d, *J* = 16.2 Hz, H-3′′′′′′), a monosubstituted benzene moiety (*δ*_H_ 7.21, overlapped, H-5′′′′′′/H-9′′′′′′; 7.29, d, 7.8, H-6′′′′′′/H-8′′′′′′; 7.31, overlapped, H-7′′′′′′) and four anomeric protons (*δ*_H_ 4.97, d, *J* = 6.6 Hz, H-1′; 5.68, d, *J* = 7.2 Hz, H-1′′; 6.04, br s, H-1′′′′; 6.13, d, *J* = 6.0 Hz, H-1′′′). The ^13^C NMR spectrum (Table 1), in combination with the HSQC data, disclosed the resonances of 67 carbons, which were sorted by ten methyls, nine methylenes, thirty-six methines, and ten quaternary carbons. The carbon signals were observed and assigned to three carbonyl groups (*δ*_C_ 167.4, C-1′′′′′′; 168.5, C-1′′′′′; 172.9, C-6′), twelve unsaturated double bonds (*δ*_C_ 119.6, C-2′′′′′′; 126.0, C-12; 128.7, C-5′′′′′′/C-9′′′′′′; 129.5, C-6′′′′′′/C-8′′′′′′; 129.7, C-2′′′′′; 130.5, C-7′′′′′′; 135.3, C-4′′′′′′; 137.1, C-3′′′′′; 144.1, C-13; 145.1, C-3′′′′′′) and four anomeric carbon signals (*δ*_C_ 101.6, C-1′′′; 102.7, C-1′′′′; 104.2, C-1′′; 167.405.8, C-1′). A detailed analysis of the 2D NMR data (Figure S2–S6) assembled the planner structure of compound **1** (Figure 2), which has a structure similar to that of the known compound floratheasaponin E, except for a *trans*-2-cinnamoyl group replacing the angeloyl group at C-22 [22].

**Figure 3:**
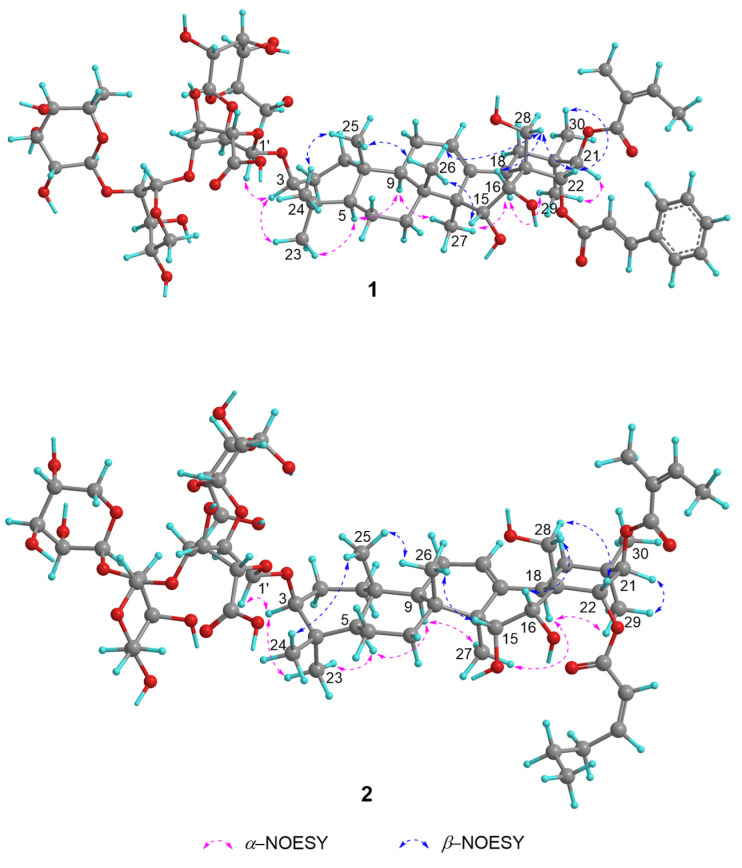
Key NOESY correlations of compounds **1** and **2**.

The NOESY cross-peaks (Figure 3, Figure S7) of H_3_-24/H_3_-25/H_3_-26/H_2_-28, H_3_-26/H-15, H_2_-28/H-16, and H_3_-30/H-22 revealed that the H_3_-24, H_3_-25, H_3_-26, H-16, H-22, H_3_-30 and C-28 bonds were directed on the identical side and were designated as being *β*-oriented. Oppositely, H-3/H-23/H-5/H-9/H_3_-27/H-18/H_3_-29/H-21 indicated that H-3, H-5, H-9, H-18, H_3_-27, H_3_-29 and H-21 should be *α*-oriented. Then, the chemical structure of **1** was established to be 15*α*-hydroxy-16*α*-hydroxy-21*β-O*-angeloyl-22*α*-*O*-*trans*-2-cinnamoyl-28-dihydroxymethylene-olean-12-ene-3*β*-*O*-[*β*-D-galactopyranosyl-(1→2)]-[*α*-L-rhamnopyranosyl-(1→2)-*α*-L-arabinopyranosyl-(1→3)]-*β*-D-glucopyranosiduronic acid, named camsinsaponin A.

Compound **2** was obtained as a white amorphous powder with a molecular formula of C_63_H_97_O_27_, as established from its HR-ESIMS negative ion at *m*/*z* 1285.6218 [M − H]^−^ (calcd for C_63_H_97_O_27_, 1285.6217). The ^1^H and ^13^C NMR spectroscopic data (Table 1) were similar to those of the previously isolated saponin floratheasaponin B [23] except for the *cis*-2-hexenoyl group being located at C-22 rather than at the angeloyl group. In a detailed analysis of the 2D NMR data (Figure S12–S15), the ^1^H−^1^H COSY correlations of H-2′′′′′′/H-3′′′′′′/H-4′′′′′′/H-5′′′′′′H-6′′′′′′, in conjunction with the HMBC correlation of H-2′′′′′′/C-1′′′′′′, suggested the presence of a *cis*-2-hexenoyl group. Furthermore, the HMBC correlation from H-22 (*δ*_H_ 6.29, d, *J* = 10.2 Hz) to C-1′′′′′′ (*δ*_C_ 166.8) suggested that the *cis*-2-hexenoyl group was located at C-22 (Figure 2). Consequently, the structure of **2** was determined to be 15*α*-hydroxy-16*α*-hydroxy-21*β-O*-angeloyl-22*α*-*O*-*cis*-2-hexenoyl-28-dihydroxymethylene-olean-12-ene-3*β*-*O*-[*β*-D-galactopyranosyl-(1→2)]-[*β*-D-xylopyranosyl-(1→2)-*α*-L-arabinopyranosyl-(1→3)]-*β*-D-glucopyranosiduronic acid, named camsinsaponin B.

The known compounds (**3** and **4**) were identified by comparing their ^1^H and ^13^C NMR and HR-ESIMS data with those in the literature (Figure S17–S22). The known compounds **3** and **4** were ascertained as floratheasaponin B and floratheasaponin C, respectively [23]. Interestingly, compounds **1** and **2** were isolated and identified as undescribed compounds from *C. sinensis* flowers. Notably, compound **2** featured a *cis*-hexenoic acid side chain at the C-22, a structural characteristic that significantly differed from those of the known compounds **3** and **4**. Specifically, the *cis*-hexenoic acid side chain had not been previously reported in studies of *C. sinensis* flower saponins, further highlighting the structural novelty of **2**. The uniqueness of **1** was reflected in two places: first, the side chain at its C-22 position differs from those of **2**–**4**; second, its sugar moiety also exhibited significant differences. As shown in Figure **1**, the R_1_ group of **1** was rhamnose, whereas the R_1_ groups of **2**–**4** were xylose.

### 2.2. Antifungal Activities of Compounds 1–4

The isolated compounds **1**–**4** were tested against three *Candida* species (*C. albicans* ATCC14053, *C. glabrata* ATCC2001 and *C. tropicalis* ATCC13803) using the broth microdilution method [14,24]. Fluconazole was used as a positive control. The antifungal activity results (Table 2) showed that compounds **1**–**4** displayed significant antifungal activity, especially for *C. albicans*. Compounds **1** and **2**, their MIC (minimum inhibitory concentration) values against *C. albicans* were less than 10 μM (7.81 μM, 5.06 μM), and the inhibitory effects were close to those of the positive control, fluconazole (4.25 μM). Furthermore, the inhibitory elements of compounds **1**–**4** on *C. glabrata* were better than those of fluconazole. Previous studies have shown that theasaponin E1 and assamsaponin A, isolated from *C. sinensis* seeds, significantly enhance the cell membrane permeability and disrupt the integrity of *C. albicans* cells, probably through interactions with membrane-bound sterols [14].

Further investigation into the structure–activity relationship (SAR) revealed that compounds **1** and **2**, despite differing in their sugar moieties and the side-chain groups at the C-22 position, displayed comparable antifungal activities. This similarity limited the scope for a detailed SAR analysis based on these differences. Moreover, compounds **2**, **3** and **4** possessed identical sugar chain structures but differed in the side-chain groups at the C-22 position. The MIC test results revealed that compound **2** exhibited superior inhibitory effects against the three *Candida* species compared to compounds **3** and **4**, suggesting that the *cis*-hexenoic acid moiety at the C-22 position plays a more significant role in antifungal activity. Overall, compounds **1** and **2** showed stronger inhibitory effects against the three *Candida* species than compounds **3** and **4**. Through SAR analysis, it was concluded that the acylation of different organic acid groups at the C-22 position significantly influences the antifungal efficacy against *Candida* species. Currently, we have isolated only four saponin compounds from the *C. sinensis* flowers, which is a relatively limited number. In order to conduct a more comprehensive analysis of the structure–activity relationship, it is necessary to isolate more saponins and evaluate their biological activities in future work.

## 3. Materials and Methods

### 3.1. General Experimental Procedures

The IR spectra of compounds **1** and **2** were measured using a Nicolet 8700 FT-IR spectrophotometer (Thermo Scientific Instrument Co., Waltham, MA, USA). An Agilent 1260 HPLC system equipped with a photodiode detector array (PDA) coupled to a 6530 time-of-flight (TOF) mass spectrometer with an electrospray ionization (ESI) source (Agilent Inc., Santa Clara, CA, USA) was employed to acquire ion fragment information on **1**–**4** in negative mode. The NMR spectra of **1**–**4**, including the 1D (^1^H, ^13^C NMR) and 2D (^1^H−^1^H COSY, HSQC, HMBC, and NOESY) spectra, were recorded on an Agilent DD2 600 (Agilent Technologies, Santa Clara, CA, USA), with pyridine-*d*_5_ being used as the solvent. For column chromatography, silica gel (100–200 mesh/200–300 mesh, Qingdao Haiyang Chemical Co., Ltd., Qingdao, China) and ODS gel (50 μm, YMC Co., Ltd., Kyoto, Japan) were utilized. Semipreparative HPLC was performed on a Shimadzu Essentia LC-20 system (UV detector: 210 and 254 nm) with a YMC-Pack ODS-A column (250 mm × 10 mm, 10 μm, YMC Co., Japan). A TLC analysis was conducted using precoated silica gel GF254 plates (Qingdao Haiyang Chemical Co., Ltd., China).

### 3.2. Plant Material

The flowers of the *C. sinensis* cultivar ‘Baiye 1’ were collected from the tea plantation of Anhui Agricultural University in Hefei, Hefei, Anhui Province, China.

### 3.3. Extraction and Isolation

The *C. sinensis* flowers (1.5 kg) were crushed into powder and extracted three times with 70% ethyl alcohol (EtOH) (3 × 10 L) at 60 °C under reflux. The extract was subjected to reduced pressure evaporation to obtain an EtOH concentrated solution (0.4 kg). Then, the concentrated solution was extracted successively with petroleum ether (PE), ethyl acetate (EtOAc) and *n*-butyl alcohol (*n*-BuOH). The *n*-BuOH fraction (150 g) was subjected to silica gel (100–200 mesh) CC (70 mm × 1000 mm) and then eluted with stepwise gradients of EtOAc/MeOH (10:1, 8:1, 6:1, 5:1, 4:1, 3:1, 2:1, 1:1, 0:1, *v*/*v*) to give fractions A–H. Fraction G (8.0 g) was separated on an ODS gel column (MeOH/H_2_O, 30:70–100:0, *v*/*v*) to give twelve fractions (Frs. G1–G12). Fraction G4 (1.2 g) was purified by semipreparative HPLC [YMC-Pack ODS-A, CH_3_CN-0.3% aqueous HCOOH (38:62, *v*/*v*), 3.0 mL/min] to generate compounds **1** (*t*_R_ = 26.0 min, 21.5 mg), **2** (*t*_R_ = 29.0 min, 24.0 mg). Fraction G7 (0.6 g) was purified by semipreparative HPLC [YMC-Pack ODS-A, CH_3_CN-0.3% aqueous HCOOH (40:60, *v*/*v*), 3.0 mL/min] to afford **3** (*t*_R_ = 31.0 min, 5.6 mg). Fraction G9 (0.76 g) was purified by semipreparative HPLC [YMC-Pack ODS-A, CH_3_CN-0.3% aqueous HCOOH (42:58, *v*/*v*), 3.0 mL/min] to obtain **4** (*t*_R_ = 27.0 min, 7.2 mg).

Camsinsaponin A (**1**): white amorphous powder; UV (MeOH) *λ*_max_ (log *ε*): 214 (4.22), 279 (4.31) nm; IR (KBr) *ν*_max_: 3431, 2929, 1708, 1633, 1451, 1389, 1306, 1235, 1161, 1075, 1047, 769, 535 cm^−1^ (Figure S8); negative HR-ESIMS: *m*/*z* 1333.6220 [M − H]^−^ (calcd. for C_67_H_97_O_27_, 1233.6217) (Figure S1); ^1^H and ^13^C NMR data are provided in Table 1.

Camsinsaponin B (**2**): white amorphous powder; UV (MeOH) *λ*_max_ (log *ε*): 216 (4.27) nm; IR (KBr) *ν*_max_: 3427, 2959, 1707, 1632, 1415, 1388, 1305, 1236, 1162, 1077, 1046, 535 cm^−1^ (Figure S16); negative HR-ESIMS: *m*/*z* 1285.6218 [M − H]^−^ (calcd for C_63_H_97_O_27_, 1285.6217) (Figure S9); ^1^H and ^13^C NMR data are provided in Table 1.

### 3.4. Antifungal Activity Assay

The antifungal activity of the compounds was assessed using a broth microdilution method following the procedure outlined by the Clinical and Laboratory Standards Institute (CLSI)), with minor modifications [14,24]. The strains of *C. albicans* ATCC14053, *C. glabrata* ATCC2001 and *C. tropicalis* ATCC13803 were. obtained from the First Affiliated Hospital of Anhui Medical University (Hefei, China). In brief, this method involved diluting a fungal suspension to a concentration of 1 × 10^6^ CFU/mL in RPMI 1640 medium. Subsequently, 100 μL of this diluted suspension was added to each well of a 96-well polypropylene microplate. Following this, 100 μL of aqueous solutions containing varying concentrations of compounds were introduced to achieve final concentrations ranging from 1.9 to 1000 μg/mL. Fluconazole served as the positive control, while wells containing only fungi without any compounds acted as the negative control. Additionally, wells containing solely RPMI 1640 medium were designated as blank controls. The plates were incubated at 30 °C for a duration of 24 h. The minimum inhibitory concentration (MIC) was determined visually by inspecting the wells for any visible fungal growth. Each experiment was conducted independently in triplicate to ensure both the reliability and reproducibility of results.

## 4. Conclusions

In summary, two new triterpenoid saponins named camsinsaponin A and B (**1**, **2**) and two known analogues were isolated and identified from the flowers of *C. sinensis*. Their biological activity results showed that compounds **1**–**4** exhibited significant antifungal activity. Notably, for compounds **1** and **2**, their MIC values (7.81 μM, 5.06 μM) against *C. albicans* were close to those of the positive control, fluconazole (4.25 μM). Furthermore, the inhibitory elements of compounds **1**–**4** on *C. glabrata* were better than those of fluconazole. According to the above results, we consider the saponins from *C. sinensis* flowers to have an excellent inhibitory effect on fungi, thereby indicating them as promising candidates for the future development of novel therapeutic strategies against drug-resistant infections.

## Figures and Tables

**Table 1:** ^1^H (600 MHz) and ^13^C (150 MHz) NMR data for **1** and **2** in pyridine-*d*_5_.

Position	1		2	
	*δ*_H_, Multi (*J*, Hz)	*δ*_C_	*δ*_H_, Multi (*J*, Hz)	*δ*_C_
1	1.42, m; 0.86, m	39.5	1.41, m; 0.88, m	39.4
2	2.18, m; 1.81, m	27.0	2.20, m; 1.84, m	27.0
3	3.28, dd (11.4, 4.8)	90.2	3.29, br d (8.4)	90.0
4		40.5		40.0
5	0.82, m	56.1	0.81, overlaped	56.0
6	1.61, m; 1.37, m	19.3	1.58, br d (10.8); 1.35, m	19.3
7	2.16, m; 2.08, m	37.2	2.15, br d (13.8); 2.06, br d (13.8)	37.1
8		41.9		41.9
9	1.71, m	47.6	1.71, m	47.6
10		37.4		37.4
11	1.91, m; 1.83, m	24.4	1.89, m; 1.81, m	24.4
12	5.54, br s	126.0	5.52, br s	125.9
13		144.1		144.1
14		48.3		48.2
15	4.22, m	68.0	4.19, d (10.2)	67.9
16	4.50, m	73.5	4.39, m	73.5
17		49.1		48.9
18	3.14, m	41.4	3.11, m	41.3
19	3.11, m; 1.47, m	47.4	3.09, m; 1.45, m	47.3
20		36.7		36.7
21	6.77, br d (10.2)	79.3	6.67, br d (10.2)	79.3
22	6.41, br d (10.2)	74.5	6.29, br d (10.2)	73.5
23	1.26, s	28.5	1.28, s	28.5
24	1.18, s	17.3	1.15, s	17.3
25	0.84, s	16.2	0.84, s	16.2
26	1.02, s	18.0	1.01, s	18.0
27	1.87, s	21.6	1.85, s	21.5
28	3.77, d (10.2); 3.52, d (10.2)	63.5	3.73, m; 3.45, d (10.2)	63.3
29	1.15, s	29.9	1.13, s	29.9
30	1.37, s	20.6	1.35, s	20.5
3-*O*-GlcA			3-*O*-GlcA	
1′	4.97, d (6.6)	105.8	4.94, d (7.2)	106.0
2′	4.69, m	79.8	4.67, m	79.5
3′	4.60, m	83.3	4.44, m	84.3
4′	4.61, m	71.8	4.51, m	71.6
5′	4.35, m	77.3	4.52, m	77.5
6′		172.9		173.0
2′-*O*-Gal			2′-*O*-Gal	
1′′	5.68, d (7.2)	104.2	5.75, d (7.2)	103.9
2′′	4.49, m	73.8	4.49, m	74.2
3′′	4.45, m	73.9	4.32, m	75.6
4′′	4.50, m	70.6	4.56, m	70.5
5′′	4.30, m	75.6	4.27, t (6.6)	76.8
6′′	4.52, m; 4.43, m	62.9	4.46, m	62.4
3′-*O*-Ara			3′-*O*-Ara	
1′′′	6.13, d (6.0)	101.6	5.80, d (7.2)	102.1
2′′′	4.73, m	77.4	4.60, m	82.3
3′′′	4.51, m	73.9	4.36, m	73.8
4′′′	4.28, m	69.3	4.33, m	68.7
5′′′	4.44, m; 3.90, d (10.2)	65.8	4.43, m; 3.74, t (9.6)	66.3
2′′′-*O*-Rha			2′′′-*O*-Xyl	
1′′′′	6.04, br s	102.7	5.04, d (7.2)	107.2
2′′′′	4.70, m	72.9	4.16, m	76.1
3′′′′	4.66, d (8.4)	73.1	4.04, d (7.8)	78.6
4′′′′	4.24, m	74.4	4.23, m	71.2
5′′′′	4.78, m	70.4	4.40, m; 3.52, d (10.2)	67.9
6′′′′	1.52, d (4.2)	18.8		
21-*O*-Ang			21-*O*-Ang	
1′′′′′		168.5		168.3
2′′′′′		129.7		129.5
3′′′′′	5.88, dq (7.2)	137.1	5.98, dq (7.2)	137.2
4′′′′′	2.03, overlapped	16.2	2.11, d (6.6)	16.3
5′′′′′	2.03, overlapped	21.3	2.03, s	21.4
22-*O*-Cin			22-*O*-Hex	
1′′′′′′		167.4		166.8
2′′′′′′	6.31, d (16.2)	119.6	5.54, br s	120.7
3′′′′′′	7.83, d (16.2)	145.1	6.01, m	150.6
4′′′′′′		135.3	2.72, hept (7.2); 2.64, hept (7.2)	31.4
5′′′′′′	7.21, overlapped	128.7	1.33, m	22.9
6′′′′′′	7.29, d (7.8)	129.5	0.82, t (7.2)	14.2
7′′′′′′	7.31, overlapped	130.5		
8′′′′′′	7.29, d (7.8)	129.5		
9′′′′′′	7.21, overlapped	128.7		

**Table 2:** Antifungal activities of compounds **1**–**4**.

Compounds	MIC (µM)		
	*C. a* ATCC14053	*C. g* ATCC2001	*C. t* ATCC13803
**1**	7.81 ± 3.38	13.02 ± 4.51	31.25 ± 0.00
**2**	5.06 ± 1.76	15.63 ± 0.00	26.04 ± 9.02
**3**	12.29 ± 0.00	20.47 ± 7.09	40.94 ± 14.18
**4**	12.27 ± 0.00	32.72 ± 14.16	65.41 ± 28.32
**fluconazole**	4.25 ± 0.56	42.54 ± 4.51	12.74 ± 3.90

*C. a* ATCC14053, *Candida albicans* ATCC14053; *C. g* ATCC2001, *Candida glabrata* ATCC2001; *C. t* ATCC13803, *Candida tropicalis* ATCC13803; fluconazole, used as a positive control.

## Data Availability

Data are contained within the article.

## Supplementary Materials

The following supporting information can be downloaded at https://www.mdpi.com/article/10.3390/ijms26031147/s1.
