# Supplementary material for: Two New Triterpenoid Saponins with Antifungal Activity from Camellia sinensis Flowers
Source: Int J Mol Sci. 2025 Jan 28;26(3):1147. doi: 10.3390/ijms26031147 (PMC11818768; doi:10.3390/ijms26031147)

Supporting Information

Two New Triterpenoid Saponins with Antifungal Activity from *Camellia sinensis* Flowers

Jian-Fa Zong, Zhi-Bo Hong, Zi-Hui Hu and Ru-Yan Hou *

National Key Laboratory for Tea Plant Germplasm Innovation and Resource Utilization, Anhui Provincial Key Laboratory of Food Safety Monitoring and Quality Control, Joint Research Center for Food Nutrition and Health of IHM, Anhui Agricultural University, Hefei 230036, China; zonjfa@ahau.edu.cn (J.-F.Z.); hzb1285688@163.com (Z.-B.H.); 18779650950@163.com (Z.-H.H.)

* Correspondence: hry@ahau.edu.cn

Contents

Figure S1. Negative HR-ESIMS spectrum of **1**........ 3
Figure S2. ^{1}H NMR (600 MHz, pyridine-d_5) spectrum of **1**........ 3
Figure S3. ^{13}C NMR (150 MHz, pyridine-d_5) spectrum of **1**........ 4
Figure S4. HSQC (600 MHz, pyridine-d_5) spectrum of **1**........ 4
Figure S5. ^{1}H-^{1}H COSY (600 MHz, pyridine-d_5) spectrum of **1**........ 5
Figure S6. HMBC (600 MHz, pyridine-d_5) spectrum of **1**........ 5
Figure S7. NOESY (600 MHz, pyridine-d_5) spectrum of **1**........ 6
Figure S8. IR spectrum of **1**........ 6
Figure S9. Negative HR-ESIMS spectrum of **2**........ 7
Figure S10. ^{1}H NMR (600 MHz, pyridine-d_5) spectrum of **2**........ 7
Figure S11. ^{13}C NMR (150 MHz, pyridine-d_5) spectrum of **2**........ 8
Figure S12. HSQC (600 MHz, pyridine-d_5) spectrum of **2**........ 8
Figure S13. ^{1}H-^{1}H COSY (600 MHz, pyridine-d_5) spectrum of **2**........ 9
Figure S14. HMBC (600 MHz, pyridine-d_5) spectrum of **2**........ 9
Figure S15. NOESY (600 MHz, pyridine-d_5) spectrum of **2**........ 10
Figure S16. IR spectrum of **2**........ 10
Figure S17. Negative HR-ESIMS spectrum of **3**........ 11
Figure S18. ^{1}H NMR (600 MHz, pyridine-d_5) spectrum of **3**........ 11
Figure S19. ^{13}C NMR (150 MHz, pyridine-d_5) spectrum of **3**........ 12
Figure S20. Negative HR-ESIMS spectrum of **4**........ 12
Figure S21. ^{1}H NMR (600 MHz, pyridine-d_5) spectrum of **4**........ 13
Figure S22. ^{13}C NMR (150 MHz, pyridine-d_5) spectrum of **4**........ 13

Figure S1. Negative HR-ESIMS spectrum of **1**

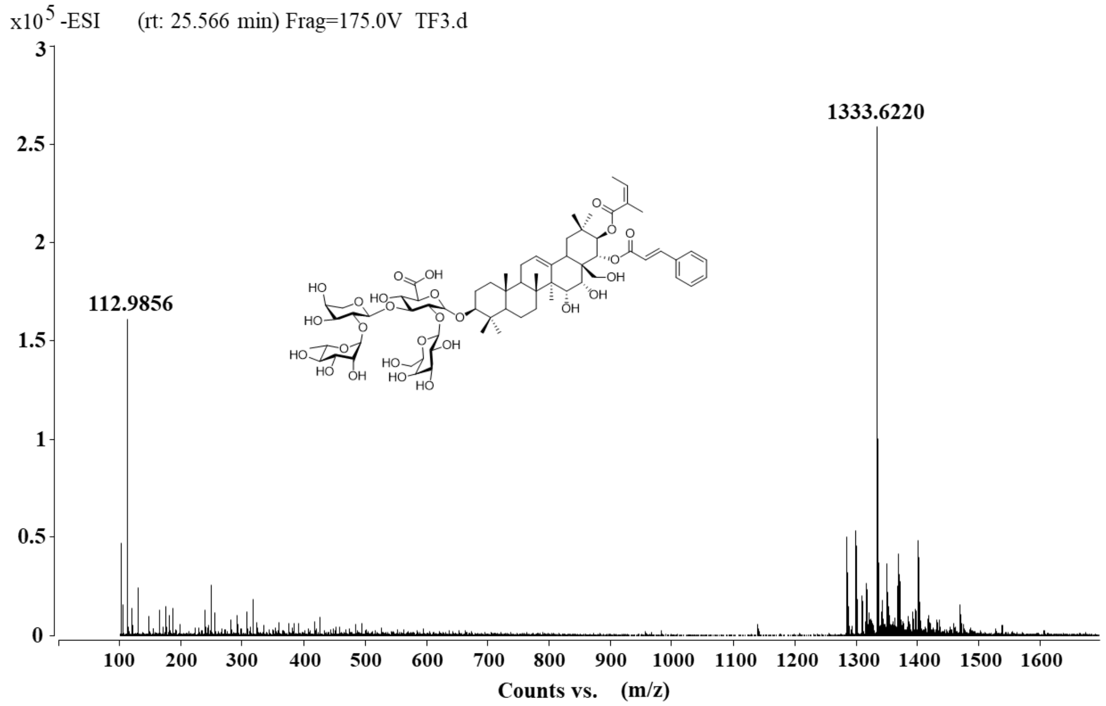

Figure S2. ^{1}H NMR (600 MHz, pyridine-d_5) spectrum of **1**

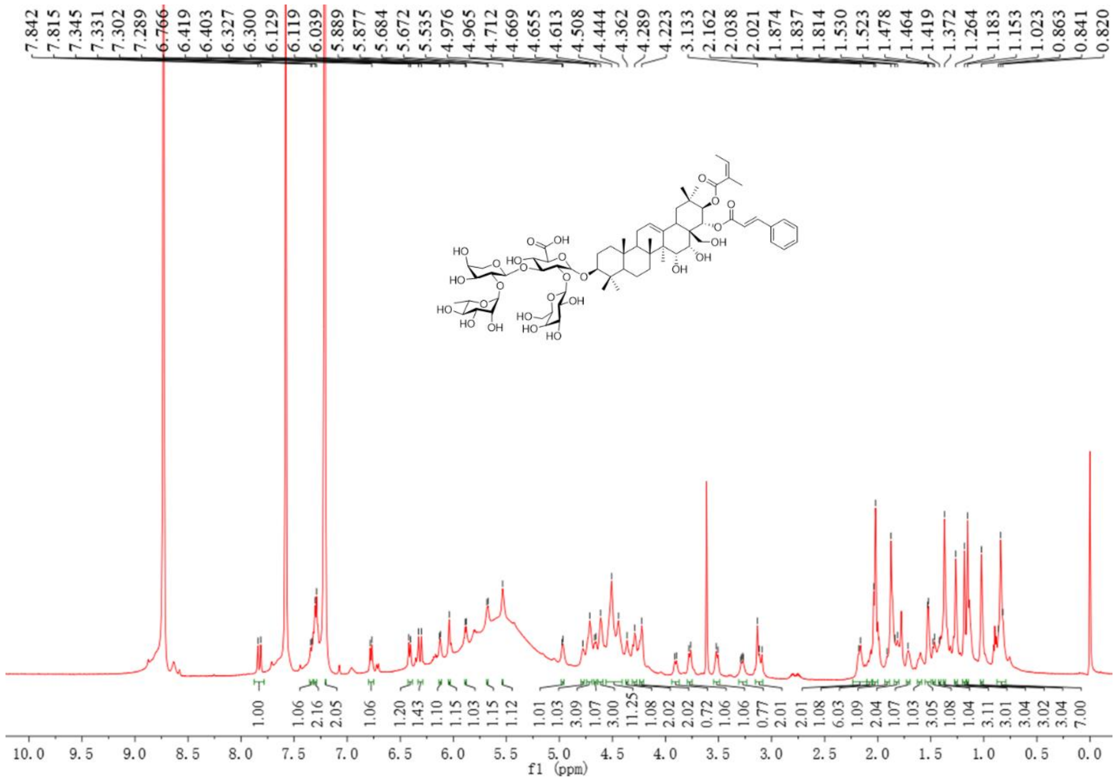

Figure S3. ^{13}C NMR (150 MHz, pyridine-d_5) spectrum of **1**

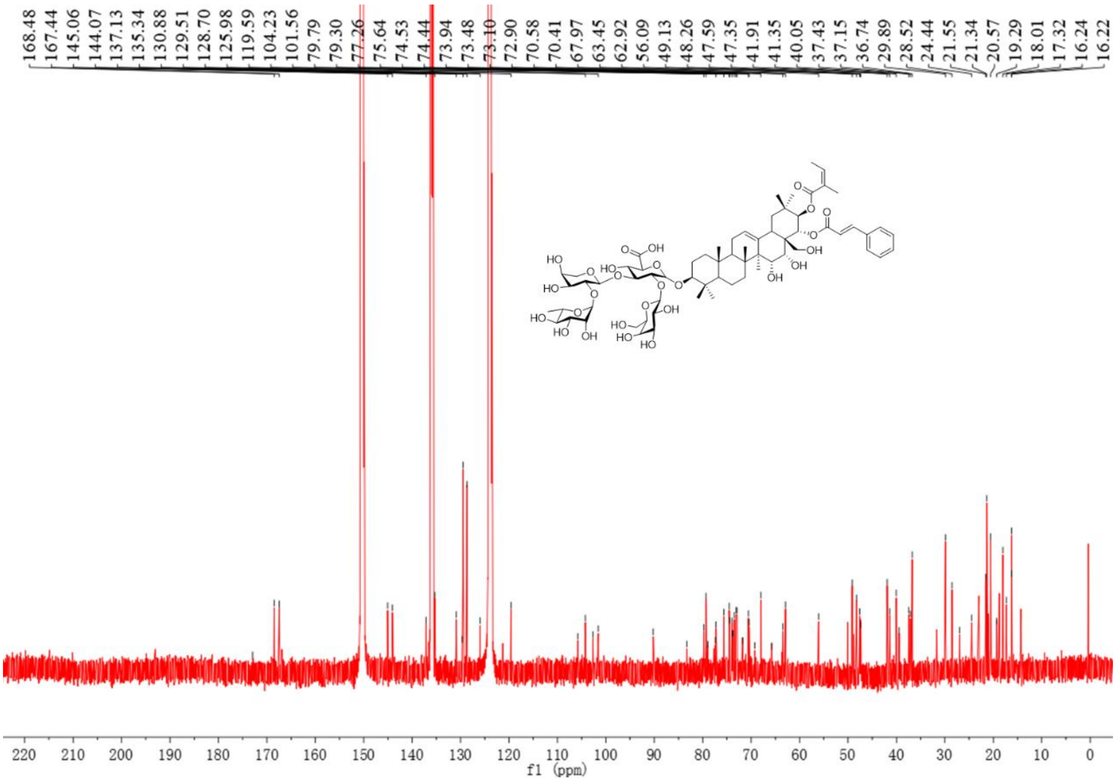

Figure S4. HSQC (600 MHz, pyridine-d_5) spectrum of **1**

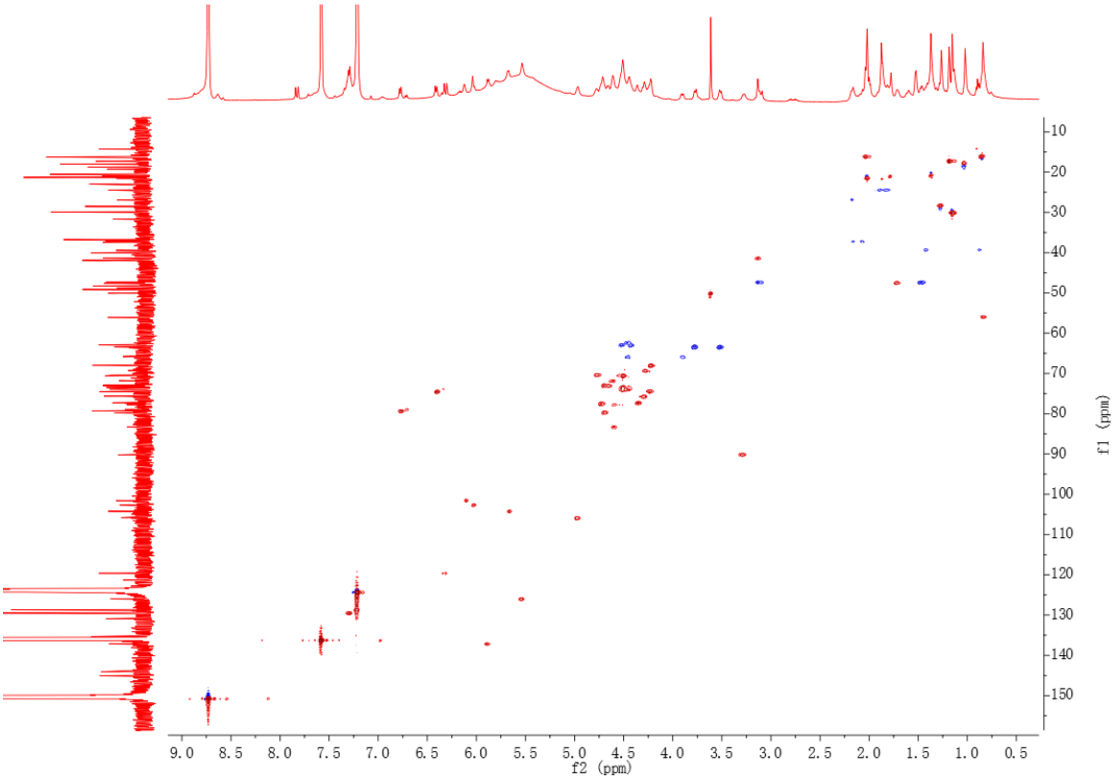

Figure S5. ^{1}H-^{1}H COSY (600 MHz, pyridine-d_5) spectrum of **1**

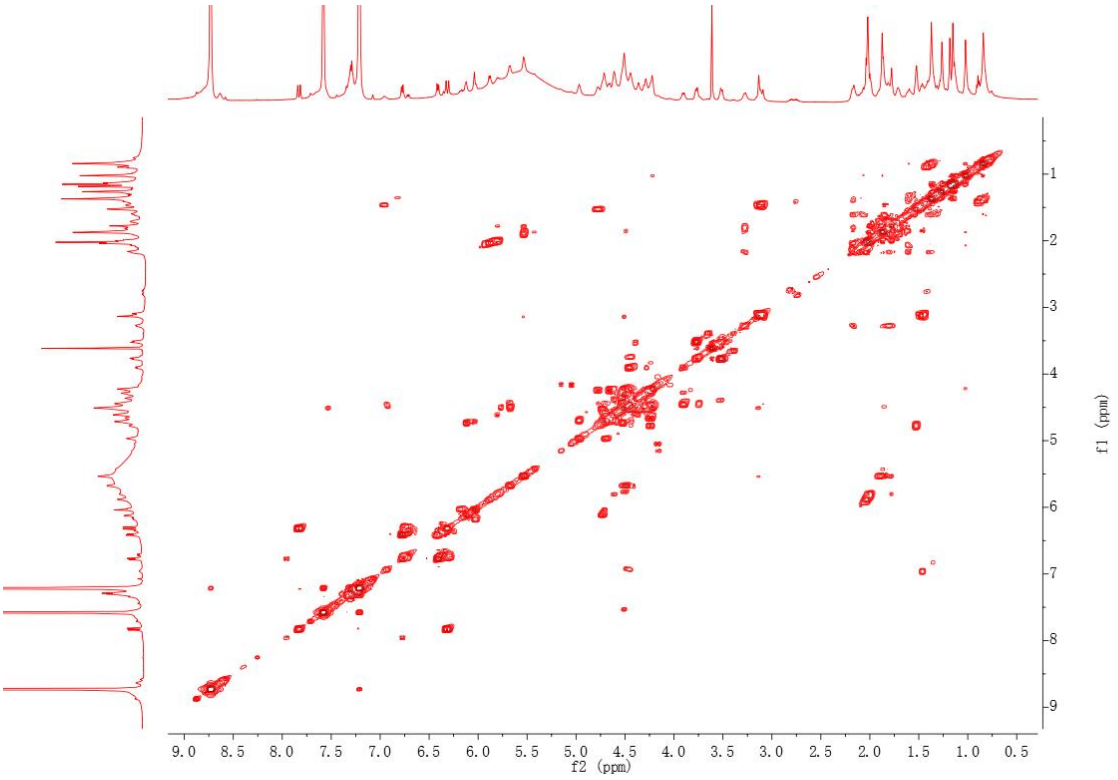

Figure S6. HMBC (600 MHz, pyridine-d_5) spectrum of **1**

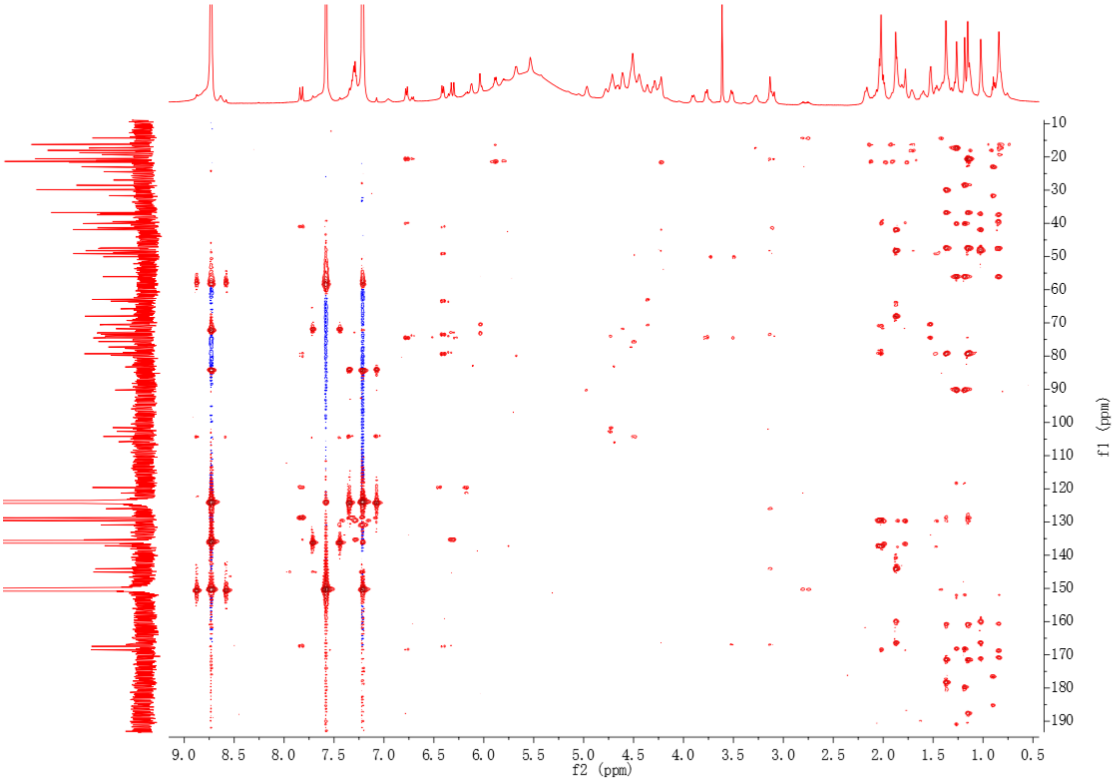

Figure S7. NOESY (600 MHz, pyridine-d_5) spectrum of **1**

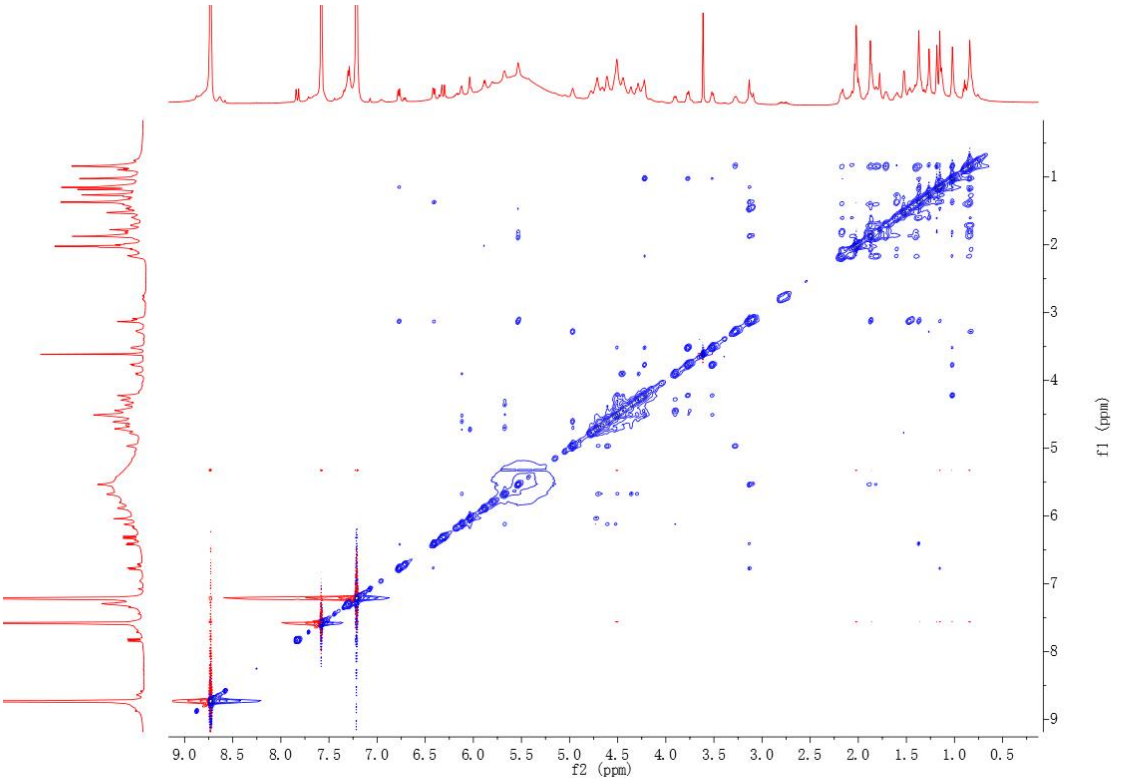

Figure S8. IR spectrum of **1**

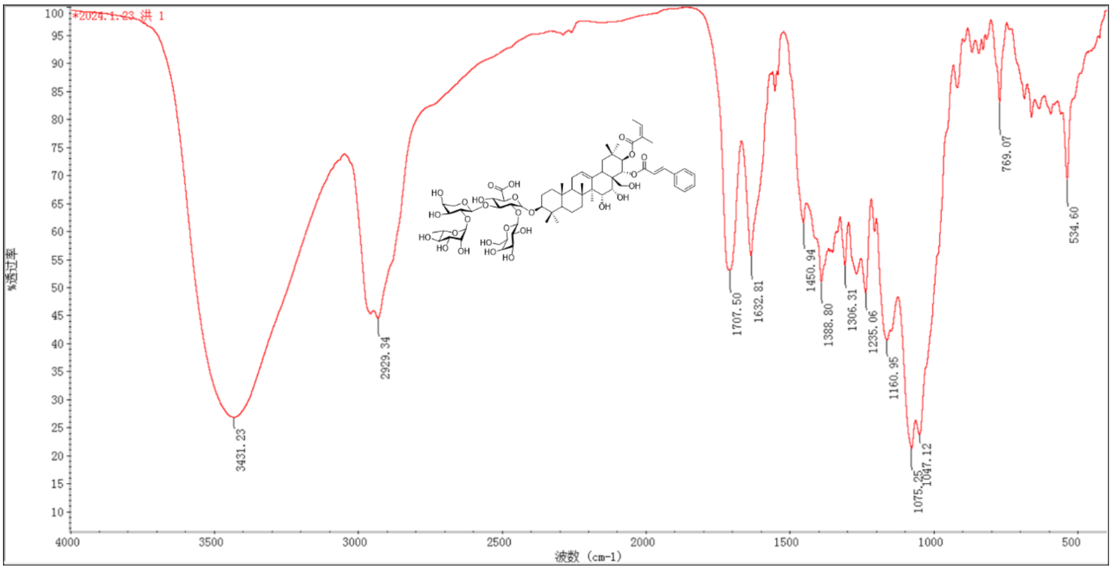

Figure S9. Negative HR-ESIMS spectrum of **2**

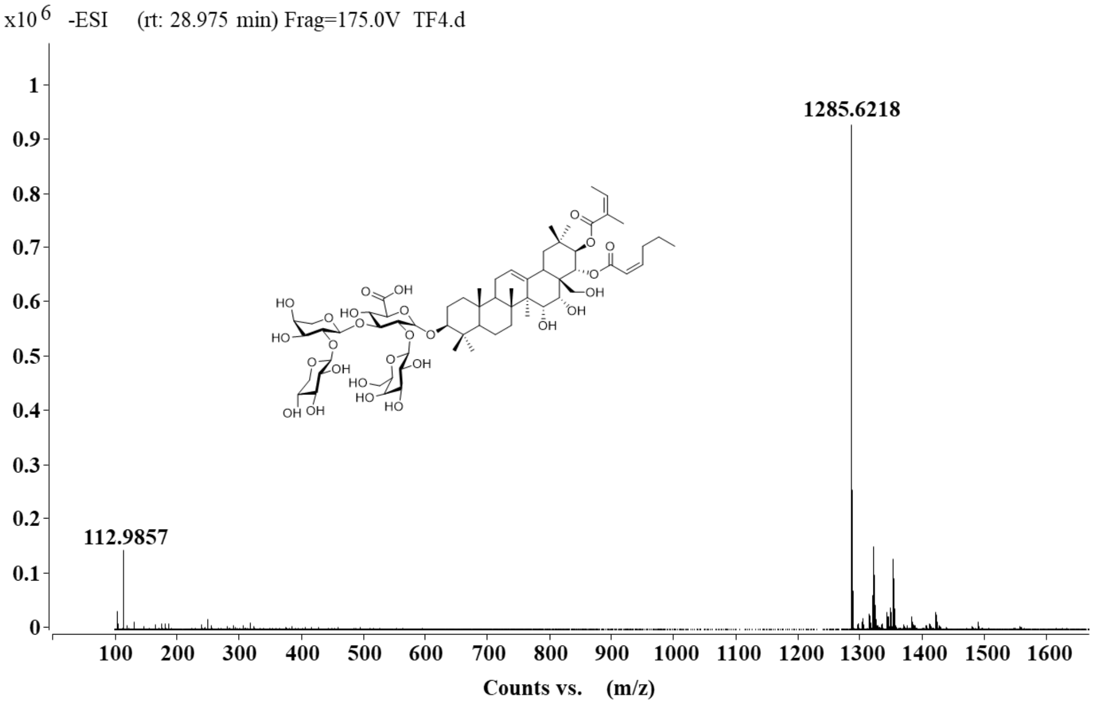

Figure S10. ^{1}H NMR (600 MHz, pyridine-d_5) spectrum of **2**

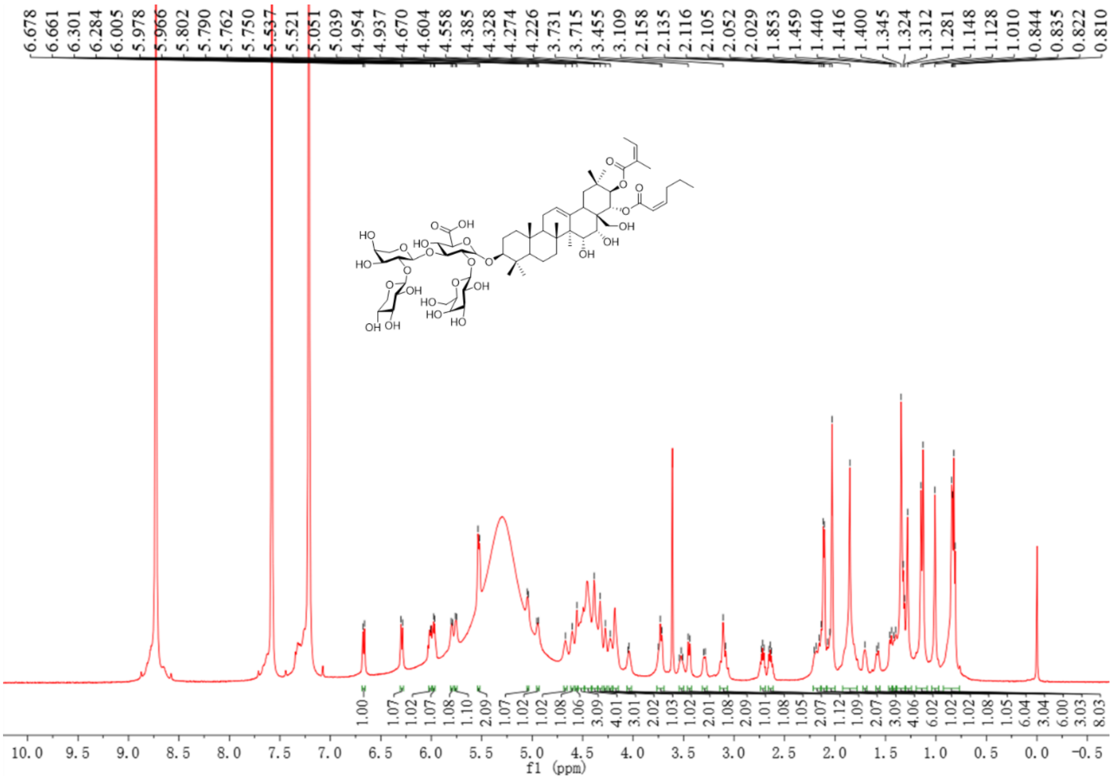

Figure S11. ^{13}C NMR (150 MHz, pyridine-d_5) spectrum of **2**

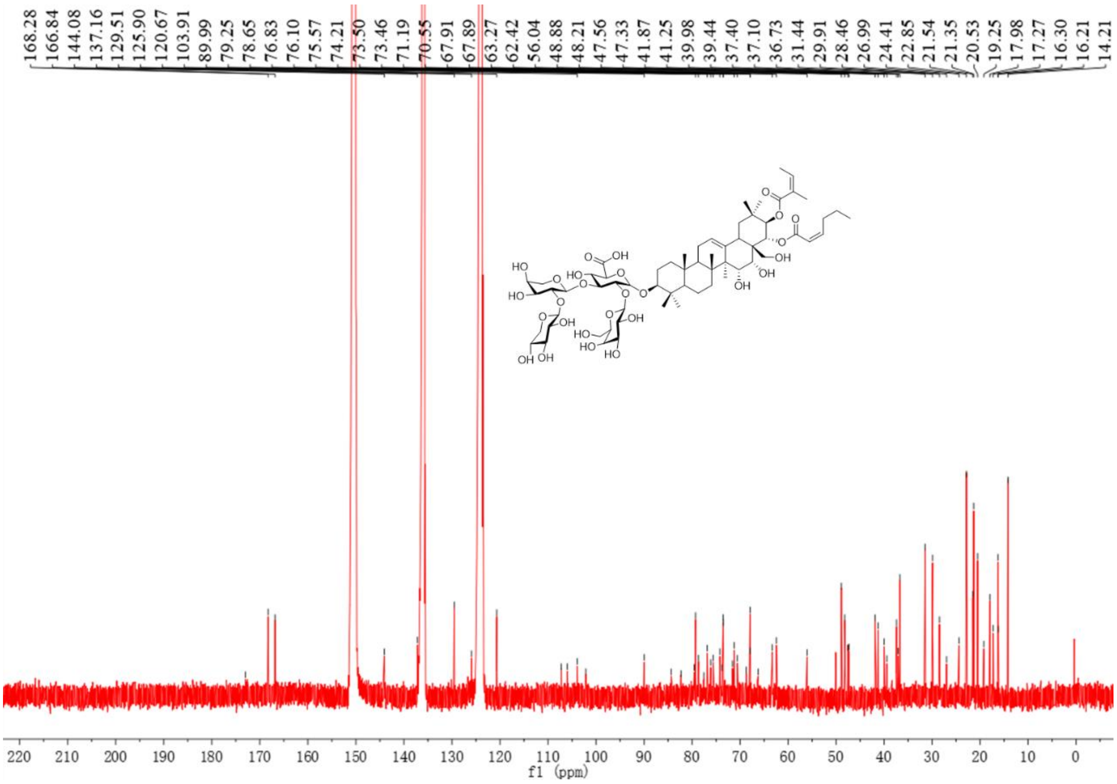

Figure S12. HSQC (600 MHz, pyridine-d_5) spectrum of **2**

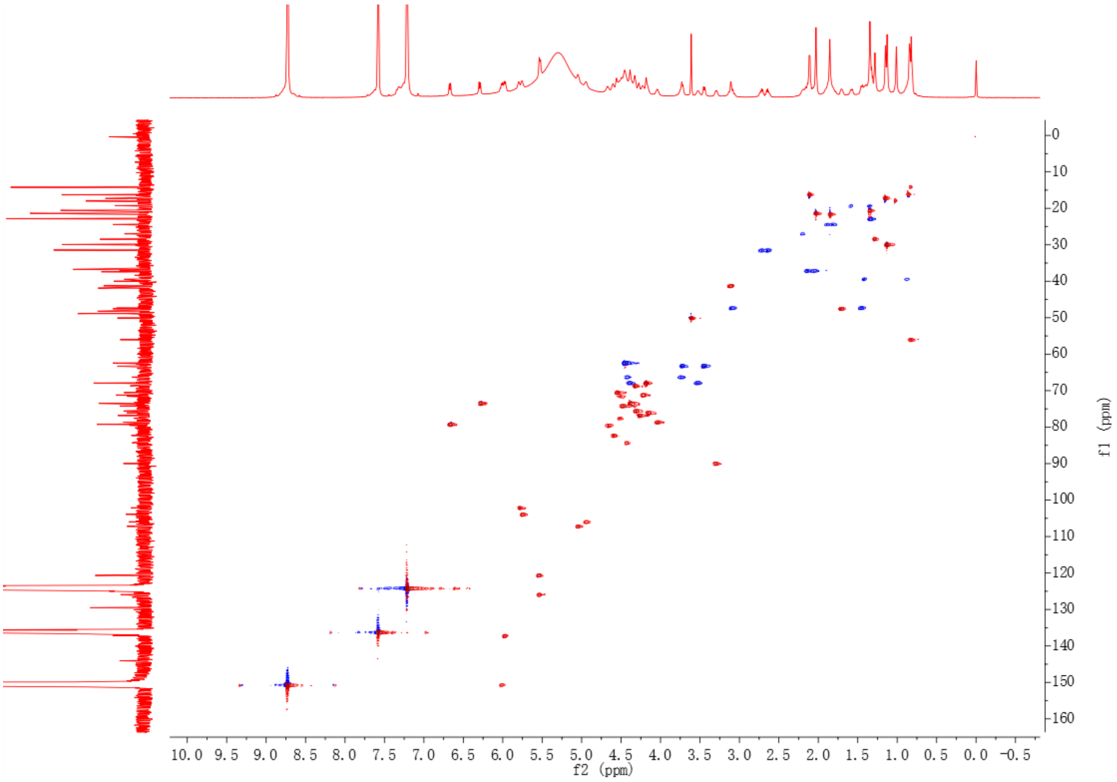

Figure S13. ^{1}H-^{1}H COSY (600 MHz, pyridine-d_5) spectrum of **2**

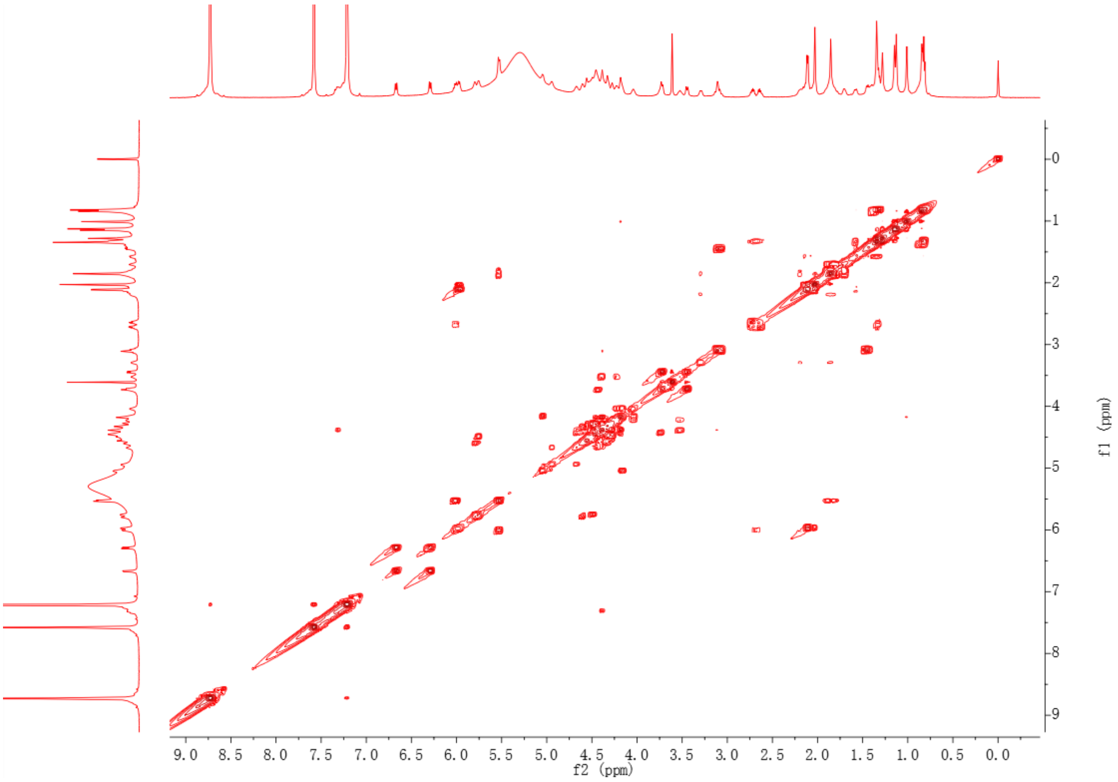

Figure S14. HMBC (600 MHz, pyridine-d_5) spectrum of **2**

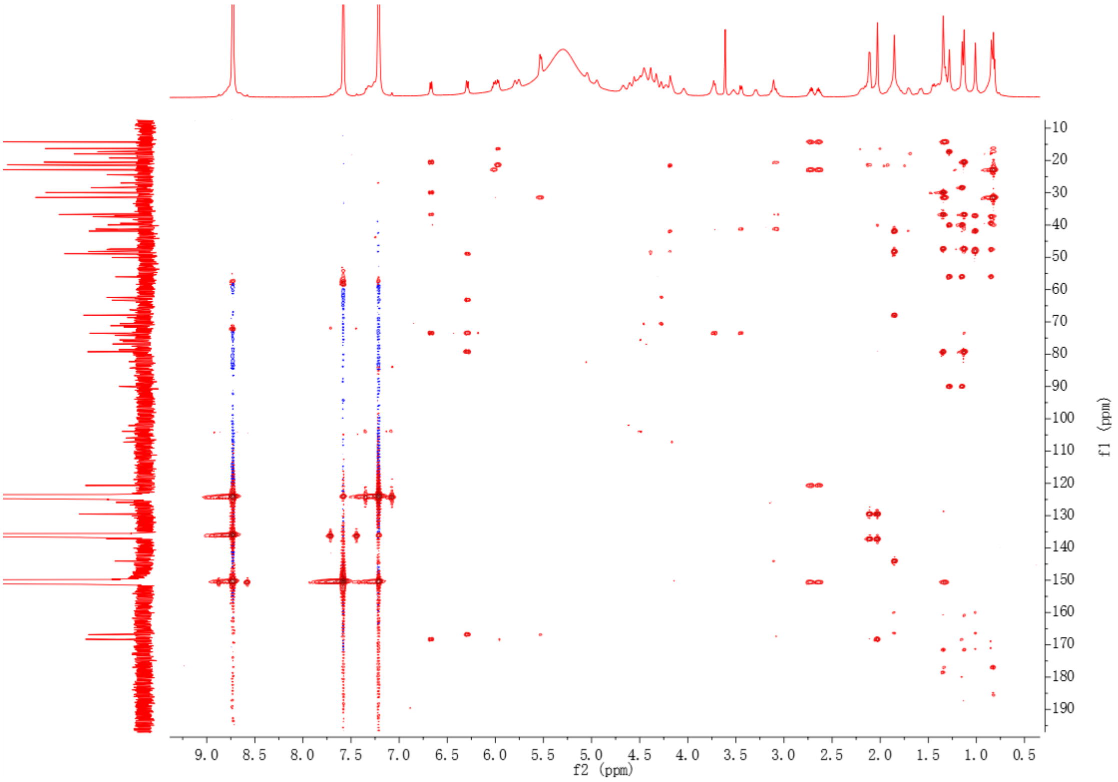

Figure S15. NOESY (600 MHz, pyridine-d_5) spectrum of **2**

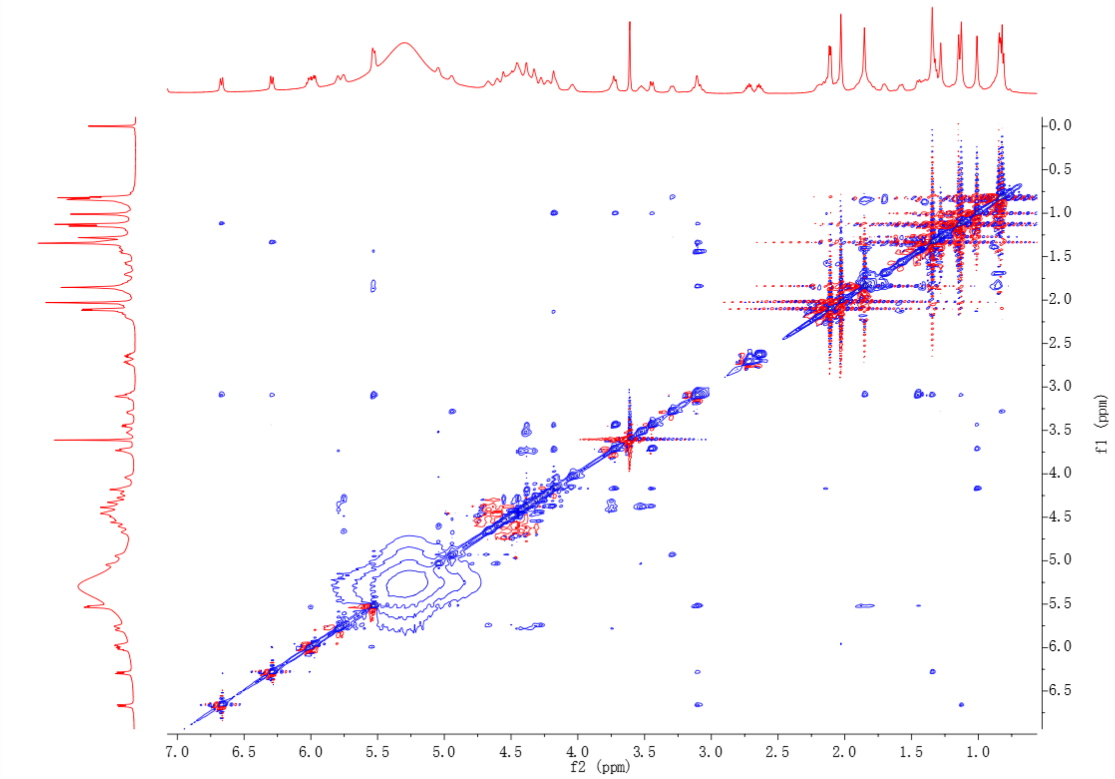

Figure S16. IR spectrum of **2**

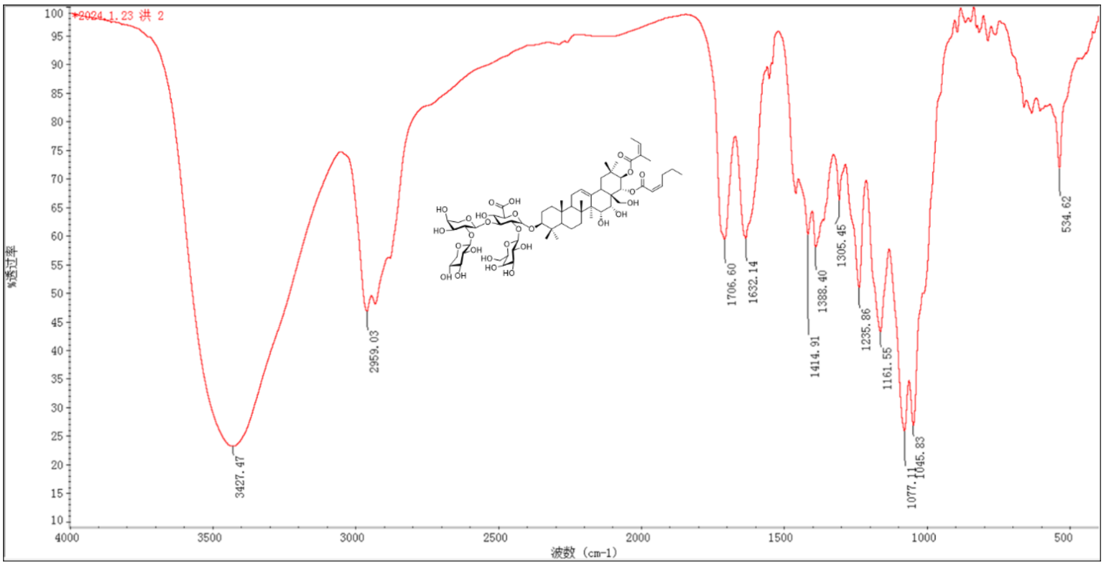

Figure S17. Negative HR-ESIMS spectrum of **3**

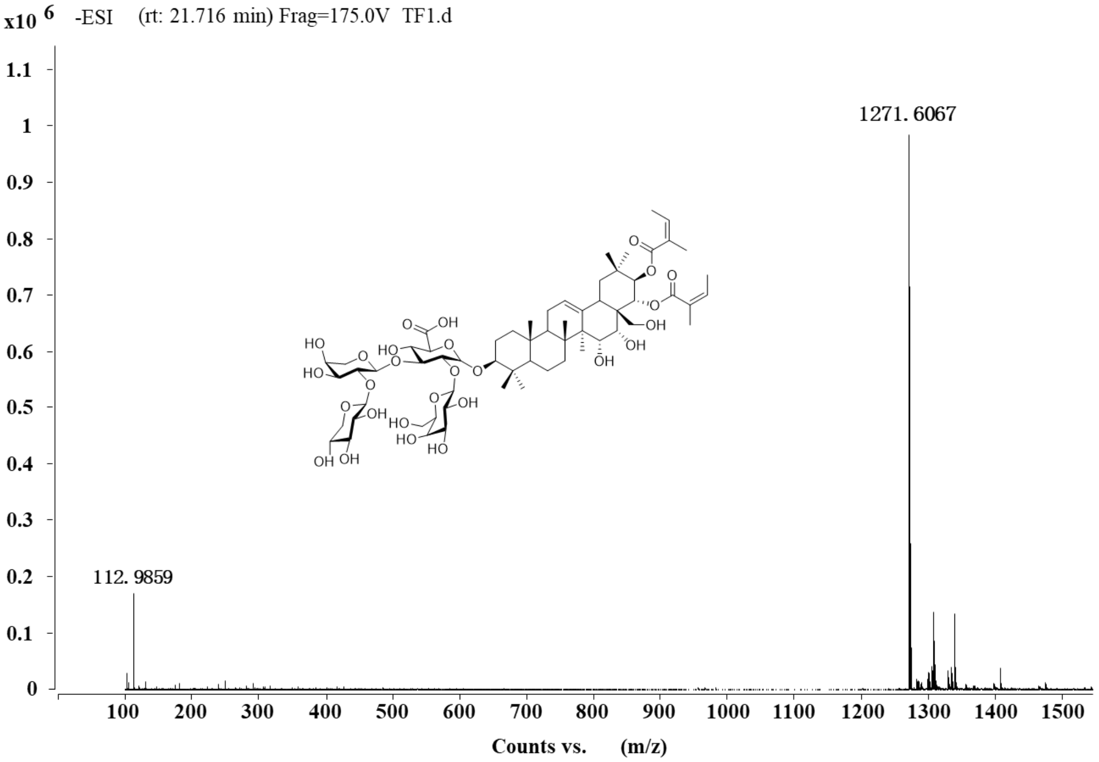

Figure S18. ^{1}H NMR (600 MHz, pyridine-d_5) spectrum of **3**

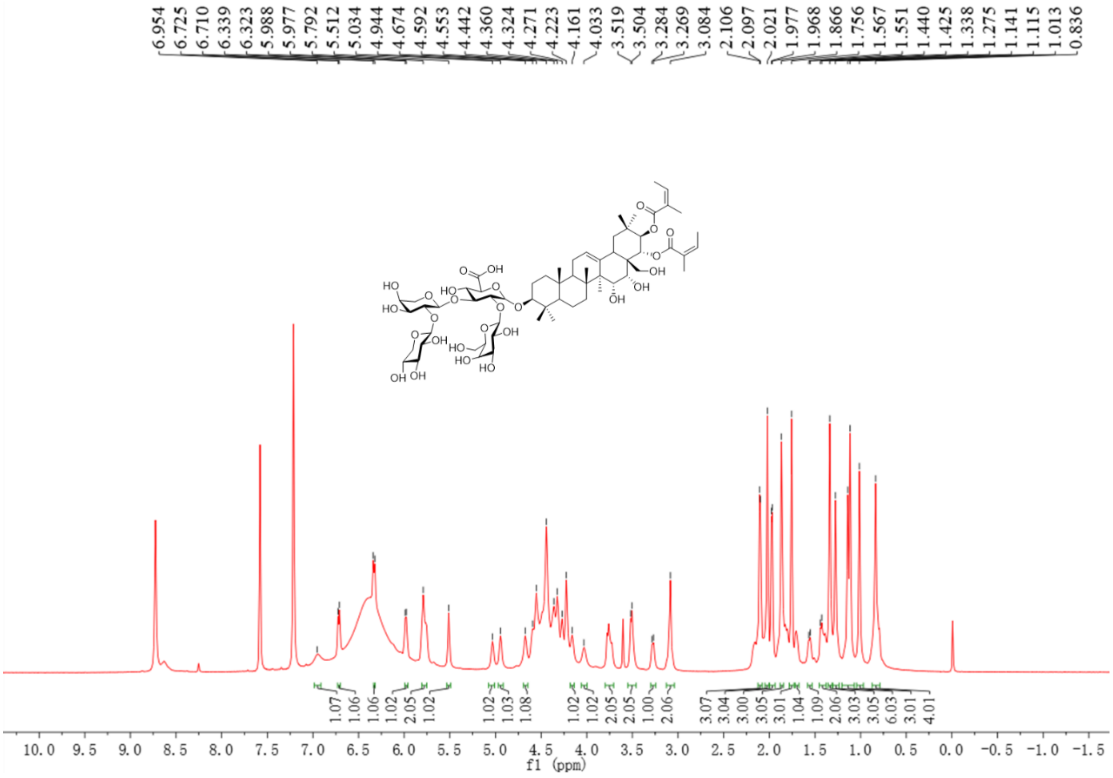

Figure S19. ^{13}C NMR (150 MHz, pyridine-d_5) spectrum of **3**

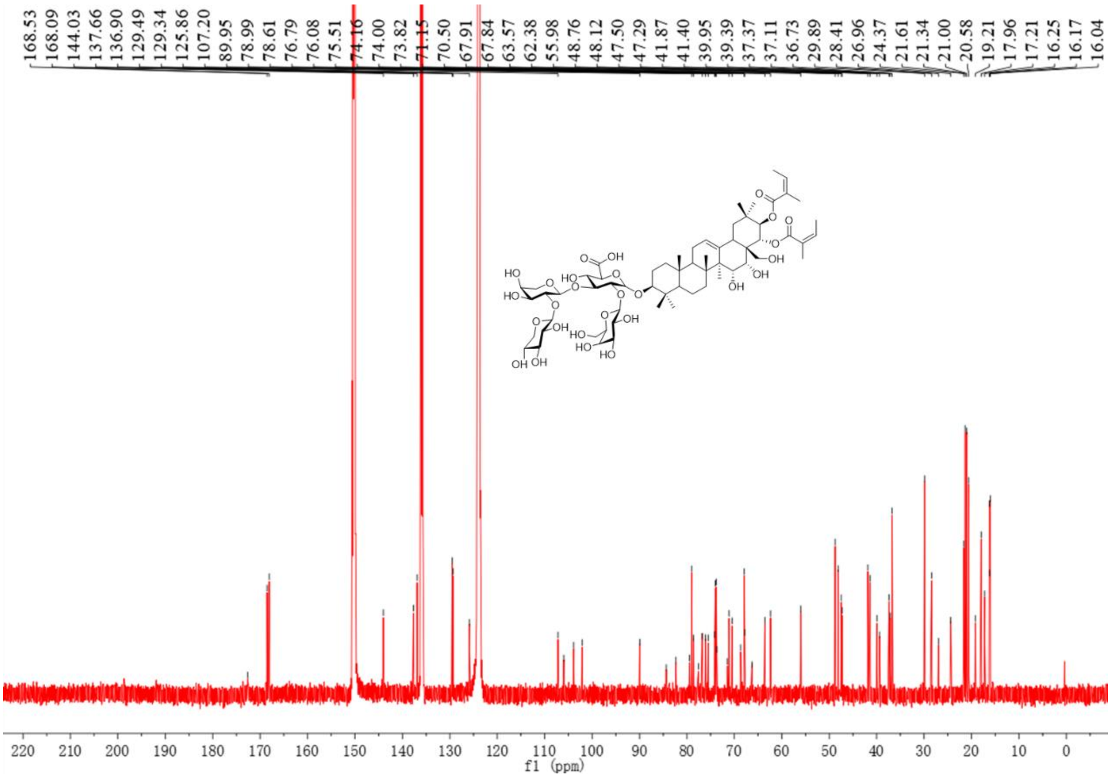

Figure S20. Negative HR-ESIMS spectrum of **4**

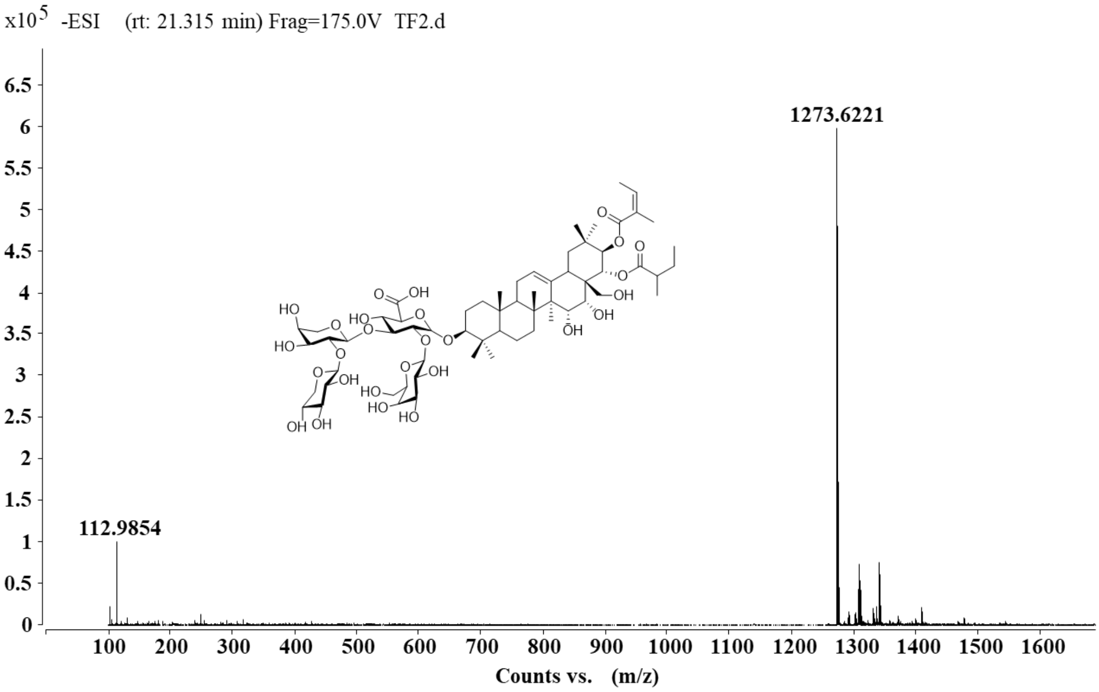

Figure S21. ^{1}H NMR (600 MHz, pyridine-d_5) spectrum of **4**

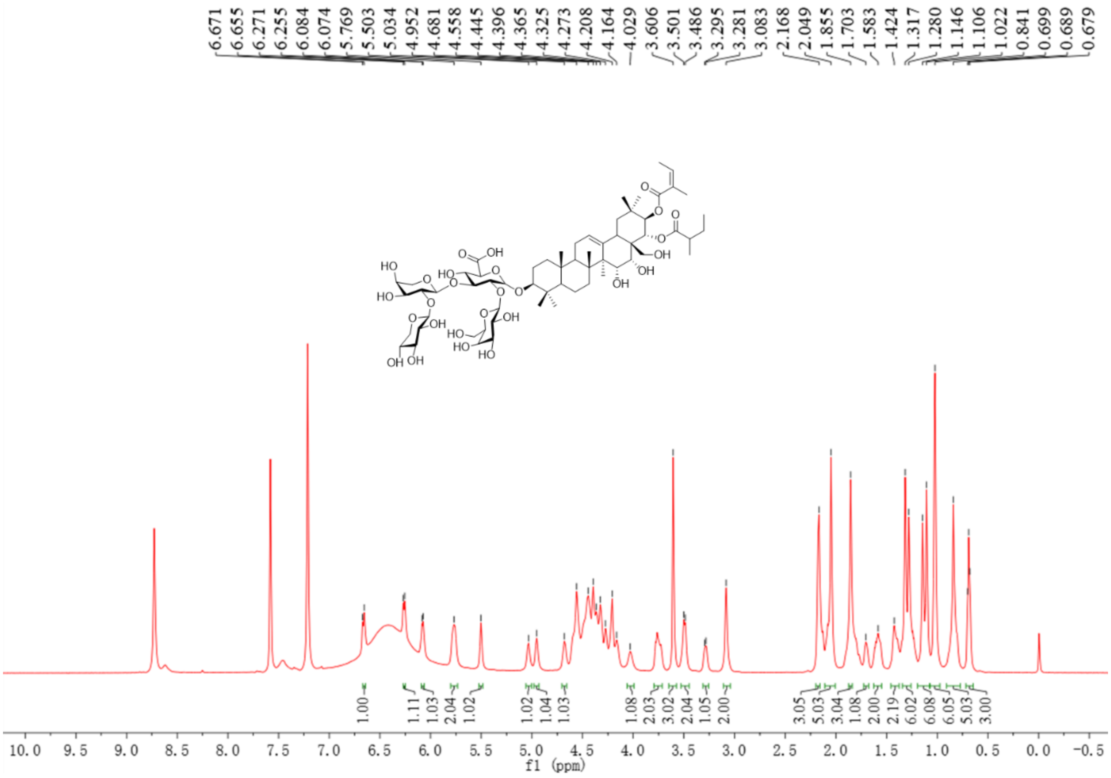

Figure S22. ^{13}C NMR (150 MHz, pyridine-d_5) spectrum of **4**

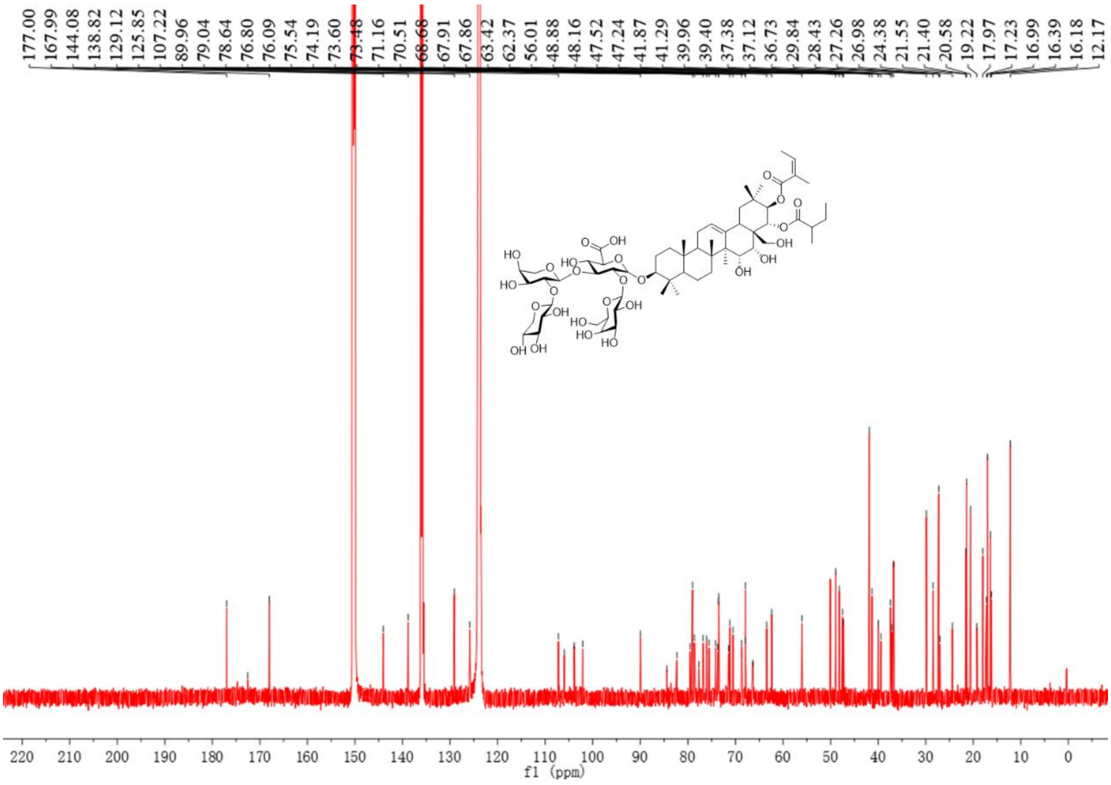

Supplement: Supplementary file 1 [file ijms-26-01147-s001.zip › ijms-3442569-supplementary.pdf]
